# O.4.1-9 vAdBeCeDa®- a multicomponent exercise programme to prevent falls and frailty in community-dwelling older adults

**DOI:** 10.1093/eurpub/ckad133.174

**Published:** 2023-09-11

**Authors:** Maja Dolenc, Tjaša Knific, Katja Tomažin

**Affiliations:** Faculty of Sport, University of Ljubljana, Slovenia; National Institute of Public Health Slovenia; maja.dolenc@fsp.uni-lj.si; Faculty of Sport, University of Ljubljana, Slovenia

## Abstract

**Purpose:**

Falls and frailty in the older adults are a considerable public health and socioeconomic problem. Physical activity has an important role in delaying degenerative ageing processes and preserving the neuromuscular system’s plasticity. Multicomponent exercise programmes could improve strength (stability), balance, mobility (flexibility) and walking speed; especially if they are carried out for at least some extend of time. Exercise programmes for fall and frailty prevention in community-dwelling older adults should include muscle performance, balance challenging and functional exercises. Slovenia has a long tradition of implementing community organized and structured exercise programmes in Health promotion centres and Healthy clubs. We are establishing new approach of frailty and falls prevention in our elderly population.

**Project description:**

With the collaboration of professionals in the field of sport recreation, health and fitness we constructed new exercise approach called vAdBeCeDa®. According to the WHO guidelines of physical activity and sedentary behaviour and the latest World guidelines for fall prevention and management for older adults, the programme is an addition to the existing Slovenian functional exercise programme called Health Promoting Sport Program ABC®. ABC programme focused especially on developing strength in fundamental movement patterns and influencing aerobic capacity. The supplement includes warm-up exercises adapted to the specific needs and functional status of the elderly, progressive balance-training programme for 12 training units and a set of static stretching exercises with support, due to poor balance. Functional and physical fitness is assed pre and post intervention with Slovenian version of senior fitness test (Jakovljević & Knific, 2015). Programme will be implemented in existing network of Health promotion centres (HPC) and in Healthy clubs (HC) all over the country, where all elderly population could practice on the Slovenian health insurance costs.

**Conclusions:**

The results from many longitudinal studies on effects of multicomponent exercise programmes are encouraging, given that maintenance of physical fitness has a successful, prolonged effect on fall and frailty prevention in older adults. All countries should pursue a lifelong physical activity for elderly, which is achievable with community approach and good collaboration between health and sport sector.

